# The changes in metabolomics profile induced by intermittent theta burst stimulation in major depressive disorder: an exploratory study

**DOI:** 10.1186/s12888-023-05044-9

**Published:** 2023-07-29

**Authors:** Xin Luo, Yuwen Zhou, Shiqi Yuan, Xiaoyu Chen, Bin Zhang

**Affiliations:** 1 Psychiatric & Psychological Neuroimage Laboratory (PsyNI Lab), The Affiliated Brain Hospital of Guangzhou Medical University, Guangzhou, China; 2 Institute of Mental Health, Tianjin Anding Hospital, Tianjin Medical University, Tianjin, China

**Keywords:** Intermittent theta burst stimulation, Depression, Metabolomics

## Abstract

**Background:**

Recently, there has been an ongoing interest in the mechanism of intermittent theta burst stimulation (iTBS) in major depressive disorder. Studying the metabolite changes induced by iTBS may help to understand the mechanism.

**Methods:**

Eleven participants with major depressive disorder received 10 days iTBS treatment. Magnetic resonance imaging (MRI) was used to target the region of the left dorsolateral prefrontal cortex (DLPFC) in each participant. We analyzed the effects of iTBS on metabolites using high-throughput profiling and assessed its impact on depressive symptoms. These analyses were considered exploratory, and no correction for multiple comparisons was applied.

**Results:**

Among the 318 measured metabolites, a significant increase in cystine, asymmetric dimethylarginine (ADMA), 1-methylhistidine, indoleacetic acid (IAA), diethanolamine (DEA), dopa, riboflavin-5′-monophosphate (FMN), and a significant decrease in alphalinolenic acid (ALA), gamma-linolenic acid (GLA), serotonin, linoleic acid (LA) (*p* < 0.05) were detected in the patients after iTBS treatment. In Pearson correlation analysis, the plasma levels of LA, FMN and ADMA at baseline were significantly related to the reduction rate of the 17‐item Hamilton Depression Rating Scale and the Patient Health Questionnaire-9 scores (*p* < 0.05).

**Conclusions:**

Our study highlights that LA, FMN, ADMA and their relationship with oxidative stress, may be key factors in the antidepressant efficacy of iTBS.

## Introduction

Transcranial magnetic stimulation (TMS), a non-invasive and well-established method for treating major depressive disorder (MDD), has garnered significant scholarly attention in recent years [1–3]. Recent studies have focused on investigating the impact of TMS on metabolite changes in the cerebrospinal fluid (CSF) [4] and utilizing magnetic resonance spectroscopy (MRS) [5] to explore alterations in brain regions associated with TMS. The findings from these studies suggest that TMS may exert its antidepressant effects by modulating metabolism.

One efficient TMS protocol, intermittent theta burst stimulation (iTBS), has gained popularity in clinical practice and research due to its shortened treatment duration and non-inferiority to the standard TMS protocol [6]. Understanding the potential mechanisms underlaying the antidepressant effects of iTBS can contribute to improved treatment approaches. Previous research has established that iTBS achieves efficient antidepressant by mimicking endogenous theta rhythms, inducing synaptic plasticity and modulating cortical excitability [7, 8]. However, much of the existing literature focus on the functions of nerve systems rather than other biological systems. Consequently, gaining a comprehensive understanding of the pathophysiology of the antidepressant mechanism mediated by iTBS remains a great challenge [9, 10].

Metabolites, as the products of gene and protein regulatory networks, reflect the pathophysiological condition of the organism [11]. Metabolomics, which can reveal the metabolic profile of diseases [12, 13], has also been widely utilized in the study of affective disorders. For example, metabolome-wide association analyses have highlighted the role of decreased blood laurylcarnitine levels and altered phosphoethanolamine levels as potential markers for MDD [14, 15]. Furthermore, Bot et al. found that depression is associated with a metabolic signature toward less high-density lipoprotein (HDL) and more very-low-density lipoprotein (VLDL) and triglycerides (TG) particles via high-throughput profiling of metabolites [16]. Another study on high-throughput targeted metabolomics profiling revealed macaques with depressive-like behaviours associated with six carbohydrate and three energy metabolism pathways [17]. Overall, these studies highlight the significant role of metabolomics in understanding the specific mechanism of depression and its treatment.

The elucidation of the metabolic profile changes in MDD after iTBS treatment is essential for comprehending the mechanism of its efficacy in depression and identifying biomarkers predictive antidepressant efficacy. A study conducted by Chou et al. examined changes in the serum inflammatory cytokine levels after 10 sessions of bilateral TBS in MDD patients [9]. They assessed a broad array of cytokines, including interleukin-6 (IL-6), C-reactive protein (CRP), and other inflammatory cytokines. However, their study did not find any significant changes in inflammatory cytokines or establish any relationship between depressive symptoms and serum cytokine levels, leading them to conclude that the antidepressant efficacy of bilateral TBS monotherapy might not operate through immune-modulating mechanisms. While Chou focuses on inflammatory cytokine levels, Stirton et al. are more concerned with oxidative stress [10]. They identified 57 oxidized phosphatidylcholines and 32 oxylipin species, which are important plasma biomarkers of oxidative stress. Notably, they found that oxidized phosphatidylcholine levels were significantly increased in patients experiencing remission from depression following iTBS treatment, suggesting a possible biomarker for predicting treatment response to iTBS. However, both Chou and Stirton's teams only analyzed one pathway or class of metabolite. The understanding of the metabolites influenced by iTBS is still in its infancy.

Metabolomics, as a widely used tool to study metabolite changes, can be classified into targeted and untargeted metabolomics [18]. Compared to the untargeted approaches, targeted metabolomics offers a high level of accurate identification and absolute quantification of metabolites of interest [19]. It also emphasizes that specifying the metabolite or metabolic pathway to be tested is a critical step in targeted approaches. Hence, most studies using targeted metabolomics tend to spotlight a specific metabolic pathway. However, focusing on one metabolic pathway may result in the loss of other metabolic information. Therefore, in the current study, we aim to address this limitation using targeted high-throughput profiling of metabolites. This approach allows the analysis of as many different metabolic pathways as possible in the same setting, maximizing the utilization of information and providing deeper insight into the therapeutic mechanisms of iTBS. Notably, no previous study has applied targeted high-throughput profiling of metabolites in iTBS treatment, enabling the measurement of different metabolites, including lipids, amino acids, and other small molecules, simultaneously [16, 17]. In this study, 41 major classes of metabolites, such as carboxylic acids and derivatives, fatty acyls, organooxygen compounds and steroids and steroid derivatives were measured by high-throughput targeted metabolomics, covering most of the common metabolites in the human body [20–22]. This comprehensive approach allows us to gain a better understanding of the antidepressant mechanism of iTBS through different metabolic pathways. We believe that the metabolomics we conducted can explore the changes involved in MDD after iTBS treatment to the best of our knowledge.

Within this context, the objective of this study is to investigate the relationship between the mechanisms of ITBS and metabolites to elucidate its antidepressant effects in MDD patients via targeted high-throughput profiling of metabolites. We collected data from MDD patients before and after iTBS, hypothesizing that the correlation between persistent improvements in depression and alterations in metabolites would show in MDD after the iTBS treatment.

## Methods

### Subjects

Patients were recruited between December 2021 and August 2022 at the Affiliated Brain Hospital of Guangzhou Medical University, Guangzhou, China, through public advertisements and psychiatric referrals. Potential participants underwent evaluation to determine if they met the inclusion criteria: (1) age between 18 and 65 years old; (2) diagnosis of MDD according to DSM-V criteria; (3) total score of at least 17 on the 17-item Hamilton Rating Scale for Depression (HAMD-17); (4) regular use for antidepressants for more than 2 weeks; (5) free from antipsychotics for more than 2 weeks.

Exclusion criteria were as follows: (1) presence of serious brain organic diseases, such as epilepsy, stroke, encephalitis, brain trauma, etc., or serious somatic diseases such as malignant tumour, acute heart failure, multiple organ failure, etc.; (2) coexistence of other psychiatric disorders, such as schizophrenia, schizoaffective disorder, bipolar disorder. (3) existence of metal implants above the chest, especially in the brain or heart. (4) women during pregnancy or lactation. (5) patients with a history of drug or alcohol abuse within the past year. (6) recent receipt of electroconvulsive therapy (ECT), or repetitive transcranial magnetic stimulation (rTMS) within the past 6 months. (7) Patients whose expressive speech difficulties hinder proper communication, understanding, following instructions, and cooperation with treatment and assessment. (8) Patients whose MRI scan images do not meet quality control standards, resulting in the inability to locate the stimulation target.

Participants were required to maintain a stable antidepressant medication regimen for 2 weeks before treatment and throughout the study. Written informed consent was obtained from all participants, and the study was approved by the ethical committee of the Affiliated Brain Hospital of Guangzhou Medical University (No. 2021–040). This study was registered with the Chinese Clinical Trial Registry (ChiCTR2100054793).

### Study procedure

After receiving ethical committee approval, eligible subjects provided written informed consent to participate in the study. All participants underwent baseline assessments of depressive symptoms using depression scales and provided baseline blood samples. Sociodemographic factors were collected, including gender, age, education and marital status, and family history. Our iTBS sessions were conducted regularly, for a total of 10 days over a 2-week period. The depression level of participants was assessed, and blood samples were collected again at the end of the treatment.

### MRI

Prior to the stimulation course, each participant underwent a structural MRI scan using the sagittal T1 weighted 3D turbo field echo (T1W 3D TFE) sequence with the following parameters: field of view = 256 × 256 mm^2^, TR/TE = 8.2/3.8 ms, view matrix = 256 × 256, slice thickness = 1 mm.

### iTBS parameters

The iTBS sessions were administered using the MAGSTIM (Magstim Co, Whitland, Dyfed, UK). The left dorsolateral prefrontal cortex (DLPFC) was targeted for stimulation, which was determined by a combination of 20 mm radius spheres centered along the left hemisphere at BA 9 (x = -36, y = 39, z = 43), BA 46 (x = -44, y = 40, z = 29), the 5-cm approach site (x = -41, y = 16, z = 54), and the F3 Beam group average stimulation site (x = -39, y = 26, z = 49) [23]. The Visor 2 system (ANT Neuro B.V.), a neuronavigation system, was used to guide the figure-of-eight coil to the left DLPFC in each session. Before each iTBS session, the system was set up by co-registering the patient’s head with their structural MRI data, enabling continuous tracking of the coil position.

A burst of 3 TMS pulses with an intensity of 100% of resting motor threshold (RMT) was applied at 50 Hz, lasting for 2 s, followed by an 8-s rest. 600 pulses were included in two sessions per day, with durations of 200 s. The treatment included a total of 20 sessions conducted over 2 weeks, administered on 5 consecutive days per week. Four patients received only one session per day.

### Measurements

#### Depression

Depression was assessed up to 3 days before treatment and 24 h after the iTBS treatment. The severity of depressive symptoms was measured using the 17‐item Hamilton Depression Rating Scale (HAMD‐17) [24] and the Patient Health Questionnaire-9 (PHQ-9) [25].

#### Metabolites

We targeted metabolites commonly involved in previous studies, such as carboxylic acids and derivatives, fatty acyls, organooxygen compounds and steroids and steroid derivatives [20–22]. Using targeted high-throughput metabolomics, we reliably quantified 318 metabolites or metabolite ratios from plasma samples. For liquid chromatography – mass spectrometry (LC–MS) analysis, the samples were re-dissolved in 100 μL of acetonitrile/water (1:1, v/v) solvent and centrifuged at 14,000 g at 4 ℃ for 15 min. Then, the supernatant was injected. The analyses were performed using an UHPLC (1290 Infinity LC, Agilent Technologies) coupled to a QTRAP MS (6500 + , Sciex) in Shanghai Applied Protein Technology Co., Ltd. The analytes were separated on HILIC (Waters UPLC BEH Amide column, 2.1 mm × 100 mm, 1.7 μm) and C18 columns (Waters UPLC BEH C18-2.1 × 100 mm, 1.7 μm). Quality control (QC) samples were included in the sample queue to evaluate the stability and repeatability of the system.

MultiQuant or Analyst was used for quantitative data processing. The QCs were processed alongside the biological samples. Metabolites in the QCs with a coefficient of variation (CV) less than 30% were denoted as reproducible measurements [26, 27].

### Statistical analysis

Statistical analysis of demographic and clinical data was performed using SPSS 25.0 (SPSS, Inc., Chicago, IL, USA) or MATLAB R2020b (The MathWorks Inc., USA). All values are presented as mean ± standard deviation (SD). The paired t-test was used to compare the pre- and post- differences in HAMD-17, PHQ-9, and metabolites. Statistical significance was set at *p* < 0.05 (2-tailed tests). In addition, we calculated the reduction rate of HAMD-17 and PHQ-9.

Pearson correlation analysis was used to evaluate the relationship between the reduction rate of the HAMD-17 and PHQ-9 scores and the plasma levels of metabolites which showed significant differences in pair t-test at baseline. The formula for calculating the rate of change of depressive scores, including HAMD-17 and PHQ-9, is shown below:

$$\mathrm{Rate}\;\mathrm{of}\;\mathrm{change}\;\mathrm{of}\;\mathrm{depressive}\;\mathrm{scores}\;=\;\left(\mathrm{pre}-\mathrm{treatment}\;\mathrm{score}\;-\;\mathrm{post}-\mathrm{treatment}\;\mathrm{score}\right)/ \left(\mathrm{pre}-\mathrm{treatment}\;\mathrm{score}\right)\times 100\% .$$

These analyses were considered exploratory, and no correction for multiple comparisons was applied.

## Results

### Participant characteristics

We enrolled 17 participants, but 5 of them dropped out. One was excluded from analyses because of its unqualified metabolite data. Therefore, data from 11 participants were analyzed (male, *n* = 5, female, *n* = 6). The mean age of the cohort was 30.18 ± 8.875 years. Two participants reported no medication usage during the study period. Four participants were taking selective serotonin reuptake inhibitors (SSRIs), while 2 participants were on serotonin-norepinephrine reuptake inhibitors (SNRIs). Additionally, 3 participants were undergoing other alternative treatment approaches.

### Antidepressant efficacy of iTBS therapy

**Fig. 1:**
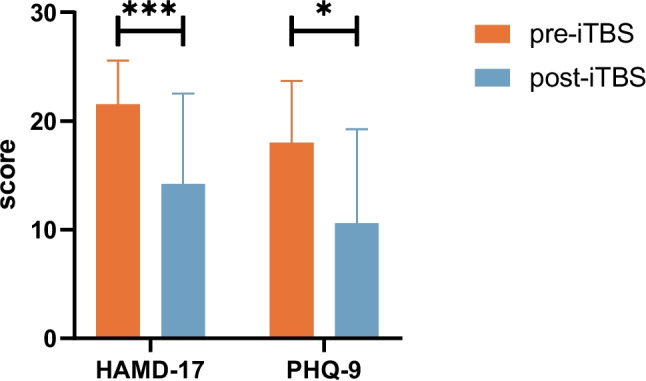
Total scores of HAMD-17 and PHQ-9 before (orange bar) and after (blue bar) iTBS treatment. The error bars indicate the standard error. *p < 0.05, ***p < 0.001

As shown in Fig. 1, the paired t-test pre-post iTBS demonstrated a statistically significant score reduction of HAMD-17 (mean pre- 21.5 ± 4.06 vs. post- iTBS 14.2 ± 8.35; *p* < 0.001) and the PHQ-9 scores (mean pre- 18.0 ± 5.69 vs. post- iTBS 10.6 ± 2.61; *p* = 0.011) after 10 days treatment of iTBS treatment.

### Changes in plasma metabolites between before and after treatment

**Fig. 2:**
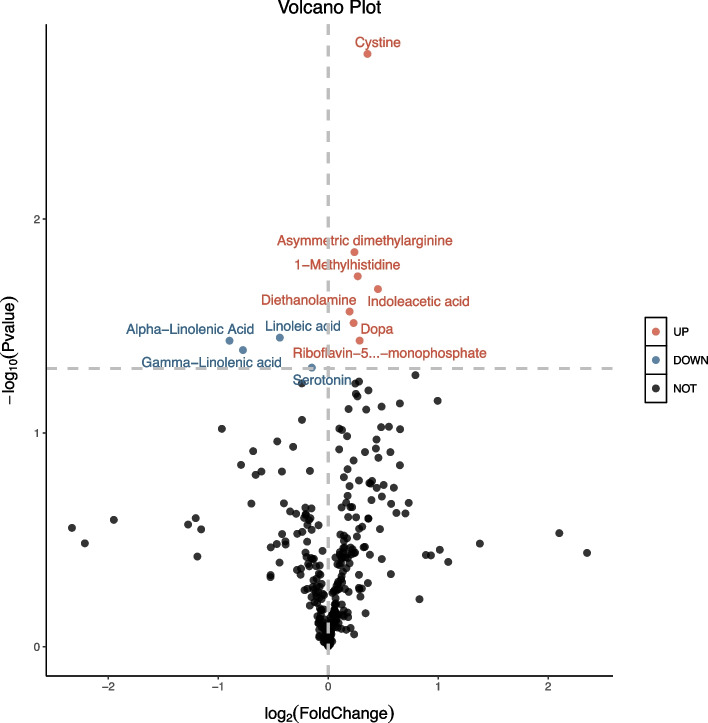
The plasma levels of 318 metabolites were detected by targeted high-throughput metabolomics profiling, presented using a volcano plot. Significantly (*p* < 0.05) changed metabolites after iTBS are shown as blue dots (downregulated) and red dots (upregulated). The X-axis represents the log2 fold change between before and after iTBS. Y-axis represents the − log10 *p*‐value of a paired t-test

From Fig. 2, it is apparent that 10 days iTBS induced metabolites changes. This includes cystine (1.23 fold, *p* = 0.002, 95% CI: 0.455 – 2.075), asymmetric dimethylarginine (ADMA) (1.18 fold, *p* = 0.014, 95% CI: 0.172 – 1.583), 1-methylhistidine (1.20 fold, *p* = 0.019, 95% CI: 0.137 – 1.527), indoleacetic acid (IAA) (1.36 fold, *p* = 0.021, 95% CI: 0.118 – 1.498), diethanolamine (DEA) (1.14 fold, *p* = 0.027, 95% CI: 0.085 – 1.446), dopa (1.17 fold, *p* = 0.030, 95% CI: 0.073 – 1.151), riboflavin-5′-monophosphate (FMN) (1.22 fold, *p* = 0.037, 95% CI: 0.048 – 1.581) increase, and linoleic acid (LA) (0.73 fold, *p* = 0.036, 95% CI: -1.387 – -0.047), alpha-linolenic acid (ALA) (0.54 fold, *p* = 0.037, 95% CI: -1.380 – -0.042), gamma-linolenic acid (GLA) (0.58 fold, *p* = 0.041, 95% CI: -1.359 – -0.028), serotonin (0.90 fold, *p* = 0.049, 95% CI: -1.804 – 0.002) decrease. Other metabolites did not significantly differ after iTBS treatment (*p* > 0.05).

### Correlation between changes in plasma metabolites and depressive symptoms

Table 1 shows that plasma LA levels at baseline were significantly negatively correlated with the rate of HAMD-17 (Pearson correlation = -0.627, *p* = 0.039) and PHQ-9 (Pearson correlation = -0.669, *p* = 0.024) reduction. A positive correlation was found between plasma ADMA level at baseline and PHQ-9 (Pearson correlation = 0.657, *p* = 0.028) reduction rate. The level of plasma FMN at baseline was positively correlated with the rate of reduction of HAMD-17 (Pearson correlation = 0.649, *p* = 0.042) and PHQ-9 (Pearson correlation = 0.855, *p* = 0.002). Baseline levels of other metabolites were not found to be significantly correlated with the rate of depressive score reduction (*p* > 0.05).

**Table 1:** Pearson correlation analysis between the plasma levels of metabolites at baseline and the reduction rate of HAMD-17 and PHQ-9

		ADMA	IAA	1-methylhistidine	ALA	Cystine	DEA	Dopa	GLA	LA	FMN	Serotonin
HAMD-17-rate	Pearson Correlation	0.553	-0.001	0.367	-0.530	0.294	-0.045	0.428	-0.479	-.627^a^	.649^a^	0.335
	*p*-value	0.078	0.998	0.267	0.094	0.380	0.896	0.217	0.136	0.039	0.042	0.417
PHQ-9-rate	Pearson Correlation	.657^a^	0.251	0.316	-0.584	0.152	0.039	0.218	-0.526	-.669^a^	.855^b^	0.067
	*p*-value	0.028	0.457	0.344	0.059	0.655	0.909	0.545	0.096	0.024	0.002	0.875

*ADMA* Asymmetric dimethylarginine, *IAA* Indoleacetic acid, *ALA* Alpha-linolenic acid, *DEA* Diethanolamine, *GLA* Gamma-linolenic acid, *LA* Linoleic acid, *FMN* Riboflavin-5′-monophosphate

^a^Correlation is significant at the 0.05 level (2-tailed)

^b^Correlation is significant at the 0.01 level (2-tailed)

## Discussion

This study aimed to examine the association of metabolites with the remission of depressive symptoms. We tracked how the emotional status, and metabolic profiles changed after 10 days of iTBS treatment by performing clinical assessments and high-throughput profiling of metabolites. We found significant reduction in depressive symptoms among patients with MDD. In addition, we observed changes in metabolites including cystine, ADMA, 1-methylhistidine, IAA, DEA, dopa, LA, FMN, ALA, GLA, and serotonin, after iTBS. The alleviation of depression after iTBS treatment is associated with a metabolic signature characterized by less LA levels and more FMN and ADMA at baseline. Based on these findings, we propose these three metabolites as potential biomarkers for predicting iTBS efficacy.

A previous study has shown that LA, an essential dietary n-6 polyunsaturated fatty acid (n-6 PUFA), is positively associated with depressive symptoms [28, 29]. Animal experiments have also demonstrated that alterations in brain lipids may contribute to the observed clinical effects of TMS [30]. Considering that iTBS is a type of TMS, it is reasonable to speculate that their underlying mechanisms could share similarities. In this study, we characterized the metabolites profile in iTBS. The level of LA was reduced on the iTBS, and lower levels of LA at baseline led to a higher reduction rate of depressive scale scores. A possible explanation for this might be that LA follows the alpha linolenic acid and linoleic acid metabolism pathways. In this pathway, LA will eventually convert to arachidonic acid (AA) which is considered a marker of oxidative stress [31, 32] and leads to a pro-inflammatory response in the cell [33]. Oxidative stress and inflammation have been shown to be important mechanisms in the pathogenesis of MDD [11, 34, 35]. Coincidentally, as another unsaturated fatty acid, the plasma level of ALA and GLA in this study were also decreased after iTBS treatment. Also, as with LA, baseline ALA and GLA levels were positively correlated with the rate of depression scale score reduction, but this correlation was not significant. LA, ALA and GLA mainly participate in the alpha linolenic acid and linoleic acid metabolism, and GLA is produced by LA catalysed by acyl-CoA 6-desaturase [36]. It is, therefore, reasonable that GLA and LA have the same trend of change. Besides, the basic chemical structures of the by-products of LA and ALA are almost similar [33]. LA and ALA are metabolized by the same set of enzymes. LA and ALA are metabolized by the same group of enzymes and the basic chemical structures of their by-products are almost similar. Thus, changes in ALA and GLA re-emphasize the role of unsaturated fatty acids in iTBS treatment. Furthermore, fasting may improve the metabolism of unsaturated fatty acids and depressive status in vivo [37]. This also suggests that future studies could classify the fat intake status of patients and investigate the efficacy of iTBS under different groups.

Another interesting finding was the increase of ADMA induced by iTBS, and the correlation between the level of ADMA at baseline and the alleviation of depression. This also accords with the earlier observations, which showed that depression severities were negatively correlated with ADMA [38]. As an endogenous of nitric oxide synthase (NOS), ADMA is also a significant metabolite in oxidative stress [39, 40]. ADMA competes with the substrate of nitric oxide synthases, impairing the L-arginine-nitric oxide pathway and leading to increased oxidative stress. These effects may have a negative impact on excitability and neurotransmission, potentially contributing to an increase in depressive symptoms [41].

Furthermore, our study revealed that iTBS treatment significantly increased FMN levels, and a significant correlation was observed between the low level of FMN at baseline and the improvement of depressive symptoms induced by iTBS. These findings corroborate previous research that associated low FMN status with depression [42, 43]. In addition, FMN’s potential to reduce oxidative stress in the brain [44] can be explained by the fact that several steps in the kynurenine pathway rely on the active form of FMN [45, 46]. The kynurenine pathway’s altered metabolism has been proposed as a link between mild chronic inflammation and depressive symptoms [47]. Inflammatory cells can generate reactive oxygen species, which may induce oxidative stress [35]. Furthermore, increasing levels of inflammation combined with oxidative stress is a ubiquitous characteristic of MDD [48].

As metabolites whose baseline plasma levels correlate with the rate of depression scale score subtraction, LA, ADMA and FMN may be biomarkers for predicting the efficacy of iTBS. Although they belong to different metabolic pathways, coincidentally their changes in this study highlight the potential role of baseline oxidative stress in predicting iTBS efficacy. The hypothesis that oxidative stresses is involved in the pathogenesis of depressive disorders is supported by many studies [34]. The brain's higher oxygen consumption and weaker antioxidative defense make it more susceptible to oxidative stress-induced damage [35]. Depression has been linked to reduced brain volume, function and neuroplasticity [49]. Studies have shown that reactive oxygen (ROS) production and reduced antioxidant defences contribute to these structural brain changes [35]. ROS can mediate lipid oxidation. Because of the high lipid content of the brain, oxidative stress is more likely to affect phospholipids in the brain, even with the blood–brain barrier in the way. Moreover, the interaction between oxidative stress and inflammation further increase the risk of depression. Depression is known to involve higher levels of inflammation compared to healthy individuals [50]. ROS and reactive nitrogen species (RNS) by immune cells and their balance play an important role in the inflammatory mechanism. Oxidative stress disrupts this balance, increasing inflammation levels and causing depressive symptoms. Greater oxidative stress-induced damage to the brain is associated with a higher probability of irreversible damage and reduced potential for treatment effectiveness. Therefore, baseline oxidative stress levels can be predictive of treatment response in MDD. Studies have indicated that iTBS can reduce oxidative stress-induced damage and alter neuroplasticity, which could explain its antidepressant efficacy in patients with depression [51, 52]. Hence, it is plausible that lower baseline damage from oxidative stress in the brain may lead to higher efficacy in iTBS.

Although this is the first study that describes metabolites profile in iTBS using targeted high-throughput metabolomics, we acknowledge some limitations in our study. First, the substantial drop-out resulting in a relatively small sample size limited our ability to precisely estimate the relationship between metabolites and depressive symptoms, even though all the effect estimates were consistent in previous studies. We acknowledg the potential for bias resulting from drop-out, which could have influenced our findings, although we cannot definitively rule out its impact. Second, the subjects in this study were primarily Asians, potentially limiting the generalizability of our findings to other ethnicities. Third, there is a potential for residual confounding due to the efficacy of drug accumulation. However, it is important to acknowledge that residual confounding may still be present due to limitations in sample size and variations in clinical treatment. Despite our efforts to mitigate confounding effects through rigorous participant selection and consistent treatment plans, the possibility of residual confounding due to unmeasured variables cannot be disregarded. Factors such as variations in disease duration, severity, or comorbidities may impact both metabolite levels and the manifestation of symptoms. Fourth, given the small sample size, the risk of type II error (false-negative error) outweighed that of type I (false-positive error), which may have led to missing meaningful group differences. As an exploratory study, no adjustments were made for multiple comparisons. Moreover, the focus of this study was on the correlation between the plasma levels of LA, ADMA and FMN at baseline and the reduction rate of depression scale score induced by iTBS, rather than significant differences in metabolites before and after treatment. Lastly, the metabolites that change during iTBS were selected in the same dataset used to correlate them with symptom reduction. This may be considered double dipping and could result in inflation of the correlation. We recognize the need for larger sample sizes in future studies to enhance the statistical power and validate our findings. Additionally, utilizing independent validation datasets would also be beneficial to confirm the robustness of the observed relationships. Despite these limitations, our study provides preliminary insights into the potential associations between the baseline metabolites levels and depressive symptom reduction following iTBS treatment.

## Conclusions

The results of this study indicate that iTBS treatment for 10 days led to changes in 11 metabolites, including cystine, ADMA, 1-methylhistidine, IAA, DEA, dopa, LA, FMN, ALA, GLA, and serotonin in individuals with MDD. Notably, the metabolites LA, ADMA, and FMN, along with their relationship with oxidative stress, may play a key role in the antidepressant efficacy of iTBS. These metabolites' baseline levels in plasma could potentially serve as biomarkers for predicting iTBS treatment response. However, it is essential to approach these results with caution due to the relatively small number of participants included in this study. Ideally, future investigations should replicate these findings in a more extensive sample of medication-free or medication-naive patients.

## Data Availability

All data generated or analyzed during this study are included in this published article.

## Abbreviations

MRI: Magnetic resonance imaging
TMS: Transcranial magnetic stimulation
MDD: Major depressive disorder
iTBS: Intermittent theta burst stimulation
HDL: High-density lipoprotein
VLDL: Very-low-density lipoprotein
TG: Triglycerides
IL-6: Interleukin-6
CRP: C-reactive protein
ECT: Electroconvulsive therapy
HAMD-17: The 17‐item Hamilton Depression Rating Scale
PHQ-9: The Patient Health Questionnaire-9
LC–MS: Liquid chromatography–mass spectrometry
SD: Standard deviation
SSRIs: Serotonin reuptake inhibitors
SNRIs: Serotonin-norepinephrine reuptake inhibitors
ADMA: Asymmetric dimethylarginine
IAA: Indoleacetic acid
DEA: Diethanolamine
FMN: Riboflavin-5′-monophosphate
LA: Linoleic acid
ALA: Alpha-linolenic acid
GLA: Gamma-linolenic acid
NOS: Nitric oxide synthase
ROS: Reactive oxygen
RNS: Reactive nitrogen species
